# Clinical features of benign paroxysmal positional vertigo

**DOI:** 10.1016/S1808-8694(15)30487-0

**Published:** 2015-10-19

**Authors:** Mariana Azevedo Caldas, Cristina Freitas Ganança, Fernando Freitas Ganança, Maurício Malavasi Ganança, Heloisa Helena Caovilla

**Affiliations:** 1 Specialization student, speech therapy course, Universidade Federal de São Paulo; 2 Adjunct substitute professor, discipline of hearing disorders, speech therapy department, Universidade Federal de São Paulo; 3 Adjunct professor, discipline of otoneurology, UNIFESP – EPM. Collaborating professor of the graduation program in vestibular rehabilitation and social inclusion, UNIBAN; 4 Full professor of otorhinolaryngology, Universidade Federal de São Paulo. Coordinator of the professional master's degree program in vestibular rehabilitation and social inclusion, Universidade Bandeirante de São Paulo; 5 Habilitation (livre-Docente) professor of otoneurology, Universidade Federal de São Paulo. Universidade Federal de São Paulo / Escola Paulista de Medicina

**Keywords:** labyrinth, nystagmus, dizziness, vertigo

## Abstract

Benign paroxysmal positioning vertigo (BPPV) is considered as the most common vestibular disease.

**Aim:**

to evaluate the age, gender, type and site of the lesion, association with other vestibular diseases, progression, and recurrence in these patients.

**Material and method:**

A retrospective series study. Data from medical reports of BPPV patients examined in series during the past six years were analyzed.

**Results:**

prevalences of BPPV were: at age 41-60 years (42.2 %); in females (62.8 %), wit nystagmus and positioning vertigo (81.3%); affecting the posterior canal (87%), unilateral (91.8 %), the right labyrinth (60.2%) – p<0.001). Due to canalithiasis (97.5%), idiopathic (74.8%), association with Menière's disease compared to other affections (55.4%); healing or recovery by means of the particle repositioning maneuver (77.9%); and possible recurrence (21.8% in a one-year follow-up period).

**Conclusion:**

BPPV is characterized by its prevalence at age 41 to 60 years, in females, with nystagmus and positioning vertigo, involving mostly the posterior canal of the right labyrinth, associated with canalithiasis or idiopathic, associated with Menière's disease compared to other affections, healing or recovery by means of particle repositioning maneuver, and possible recurrence.

## INTRODUCTION

Benign paroxysmal positional vertigo (BPPV) is the most common cause of dizziness, particularly in the elderly.^1^, ^2^, ^3^ Manifestations include brief episodes of dizziness, nausea and/or positional nystagmus associated with changes in the position of the head. These signs and symptoms are due to the undue presence in the labyrinth of calcium carbonate particles, which are statoconia originating from the utricle.^1^ Cupulolithiasis is when particles adhere to a semicircular canal cupula.^4^, ^5^ Canalithiasis is the presence of free-floating particles in the semicircular duct, rather than fixed to the cupula.^6^

BPPV is idiopathic in most cases,^7^ but may also result from cranial trauma, vertebrobasilar insufficiency, otological surgery, endolymphatic hydrops, vestibular neuritis or middle ear diseases.^8^ An association between BPPV and Ménière's disease has been reported.^9^

The features of positional nystagmus in the Dix-Hallpike maneuver^10^ or upon testing in right and left lateral decubitus indicate which labyrinth is involved (right, left or both) and which semicircular canal is affected (posterior, anterior or lateral). Positional nystagmus is generally accompanied by dizziness and/or nausea, it is paroxysmal, there may be latency, and it will fatigue upon repeated nystagmus-stimulating maneuvers.^11^

Posterior canal involvement is characterized by rotatory and upbeat vertical positional nystagmus (counterclockwise in right labyrinth lesions and clockwise in left labyrinth lesions). Anterior canal involvement is characterized by rotatory and downbeat vertical positional nystagmus (counterclockwise in right labyrinth lesions and clockwise in left labyrinth lesions). Exclusively counterclockwise or clockwise rotatory positional nystagmus suggests involvement of the vertical canal, although not defining which vertical canal is affected. In vertical canal involvement, canalithiasis is characterized by nystagmus lasting up to one minute, and cupulolithiasis is evidenced by nystagmus lasting more than one minute.^11^, ^12^

Lateral canal involvement is characterized by horizontal positional or positioning nystagmus. Horizontal positional nystagmus is geotropic when tilting the head to the right causes right horizontal nystagmus and tilting the head to the left causes left horizontal nystagmus. It is ageotropic when tilting the head to the right causes left horizontal nystagmus and tilting the head to the left causes right horizontal nystagmus. More intense geotropic nystagmus with the right ear facing downwards suggests right lateral canal canalithiasis; more intense geotropic nystagmus with the left ear facing down suggests left lateral canal canalithiasis; more intense ageotropic nystagmus with the right ear facing downwards suggests left lateral canal cupulolithiasis; more intense ageotropic nystagmus with the left ear facing downwards suggests right lateral canal cupulolithiasis.^11^, ^12^

Posterior semicircular canal involvement is the most common form in BPPV; anterior and lateral canal involvement is less frequent in this condition.^6^,^8^ Involvement may be bilateral or simultaneously affect different canals:^13^ there are other clinical variations.^14^

Particle repositioning maneuvers are the recommended therapy; these procedures are effective in most cases.^6^ 15, 16 Recurrences may require repeated therapy.^15^, ^17^, ^18^

A survey of demographic data and clinical findings of BPPV patients may help characterize the disease in our context; it is important to recognize its variations and to provide diagnostic and therapy guidelines for these cases.

The purpose of this study was to characterize BPPV patients with respects to age, gender, race, type and site of the lesions, association with other vestibular diseases, disease progression and recurrence.

## METHOD

The Research Ethics Committee of the institution in which the investigation was undertaken approved the study (protocol number 1470/06).

Data were gathered from the files of consecutive patients seen within the last six years, in whom a hypothesis of BPPV was raised by an otorhinolaryngologist.

Patients with a diagnosis of BPPV were included in our study; symptoms were episodes of rotatory dizziness upon changes in the position of the head, when lying on one or both sides, when sitting up or looking upwards. Other symptoms were nausea with or without vomiting, and other types of dizziness; positional or positioning nystagmus could also be present.^11^, ^14^, ^19^ Patients with signs or symptoms of central nervous system involvement were excluded.

Patients underwent auditory function assessments consisting of pure tone and voice audiometry and immittance testing. Vestibular function was evaluated using computerized nystagmography (Micromedical Technologies Inc. – USA) comprising the following tests: investigation of positional and positioning nystagmus, spontaneous and semispontaneous nystagmus, fixed and randomized saccadic movements, pendular tracking, optokynetic nystagmus, self-rotation of the head and caloric testing.

Investigation of vertigo and positioning nystagmus, and vertigo and positional nystagmus, were used for identifying which labyrinth and semicircular canals were involved, the pathophysiological status (canalithiasis or cupulolithiasis), and BPPV variants.

Vertigo and positioning nystagmus were investigated by applying the Dix-Hallpike maneuver using a Frenzel video (Neurograff Eletromedicina Ind. e Com. Ltda-EPT – Brasil). Each patient sat on an examination table and turned the head 45° to the side that was to be evaluated. Next, the examiner quickly brought the patient into supine while keeping the head hanging off the table and tilted at 45° to the side being evaluated for about 30 seconds. After dizziness and/or positioning nystagmus faded, the patient was returned to a sitting position and the maneuver was repeated to the other side.

Vertigo and positional nystagmus were investigated as the examiner slowly moved the patient. The sequence is from a sitting position to supine, right lateral decubitus, supine again, left lateral decubitus, supine once again and finally back to a sitting position. Next, the patient was moved from the sitting position to supine, turned the head right, then left, returned to supine and finally the sitting position. The patient would remain in each of these positions for about 30 seconds or until vertigo and/or nystagmus faded.

A statistical analysis was made of age, sex, race, type and site of lesions, association with other vestibular diseases, disease progression, and recurrence. Simple descriptive statistics for the analysis of absolute and relative frequencies was applied to characterize the sample in relation to involvement by BPPV. The chi-square test was applied to verify associations among frequencies of a sample with three categories. The chi-square test followed by the Yates correction was applied to check associations among frequencies in a two-category sample (frequency differences among pairs) when there was a difference among results (p<0.05). The significance level was 5% (a = 0.05).

## RESULTS

There were 1,271 files of patients with a diagnosis of BPPV.

Table 1 shows gender and age data of patients. BPPV predominated in females and in the 41 to 60 year age group. All patients were white.

**Table 1:** Gender and age range of 1,271 patients with benign paroxysmal positional vertigo

	Categories	Frequency	
		Absolute	Relative
		N	%
Gender	Female	798	62,8
	Male	473	37,2
Age range (years)	0-20	89	7,0
	21– 40	264	20,8
	41– 60	537	42,2
	over 60	381	30,0

BPPV with dizziness and positioning nystagmus occurred in 1,033 cases (81.3%). Canalithiasis was present in 1,007 cases (97.5%) and cupulolithiasis was present in 26 cases (2.5%). BPPV with dizziness and no positioning nystagmus was present in 238 patients (18.7%).

Table 2 show the number and percentage of BPPV cases with dizziness and positioning nystagmus in relation to the semicircular canal that was affected.

**Table 2:** Absolute and relative frequencies and a comparative analysis of unilateral or bilateral semicircular canal involvement in 1,033 benign paroxysmal positional vertigo patients.

	Involvement				
Canal	Unilateral	Bilateral	Total	Chi-square	
	N %	N %	N %	(p-value)	
Posterior	814 78,8	85 8,2	899 87,0		
Lateral	89 8,6	–	89 8,6		Posterior – Lateral < 0,001
				< 0,001	Posterior – Anterior < 0,001
Anterior	45 4,4	–	45 4,4		Lateral – Anterior = 0,002
Total	948 91,8	85 8,2	1.033 100,0		

The posterior canal was affected more frequently than the lateral and anterior canals. Unilateral posterior canal involvement was more frequent than bilateral involvement. The lateral canal was involved more often than the anterior canal. There was no bilateral involvement of anterior or lateral canals.

The disease was unilateral in 91.8% of cases, and bilateral in 8.2% of cases of BPPV with dizziness and positioning nystagmus. The disease was unilateral in 83.2% of cases of BPPV with dizziness and with no positioning nystagmus, and bilateral in 16.8% of such cases, as shown on Table 4.

**Table 4:** Absolute and relative frequencies of the behavior of unilateral involvement in 198 benign paroxysmal positional vertigo patients with dizziness and no positioning nystagmus; also a comparative analysis.

	Frequency		Chi-square
Unilateral involvement	Absolute N	Relative %	(p-value)
Right	115	58,0	
Left	83	42,0	0,027
Total	198	100,0	

Table 3, Table 4 show that the prevalence of right labyrinth involvement was significant in patients with or with no positioning nystagmus.

**Table 3:** Absolute and relative frequencies of the side in unilateral involvement of 948 benign paroxysmal positional vertigo cases with dizziness and positioning nystagmus; also a comparative analysis.

	Frequency		Chi-square
Unilateral involvement	Absolute N	Relative %	(p-value)
Right	570	60,2	
Left	378	39,8	< 0,001
Total	948	100,0	

BPPV was considered as idiopathic in 950 cases (74.8%). The etiology was cranial trauma In 191 cases (15.0%).

Table 5 shows the prevalence of associated vestibular diseases or otological conditions. BPPV secondary to other vestibular diseases or otological conditions was encountered in 130 cases (10.2%). BPPV was associated with Ménière's disease in 72 (5.7%) of 1,271 BPPV cases; with vestibular neuritis in 38 cases (3.0%); with vertebrobasilar insufficiency in 14 cases (1.1%); and with other otological conditions in 6 cases (0.5%). The prevalence of their association with BPPV was varied widely. Ménière's disease associated with BPPV was more frequent that BPPV associated with vestibular neuritis, vertebrobasilar insufficiency, chronic otitis media, and following otological surgery.

**Table 5:** Vestibular disease or otological conditions associated with benign paroxysmal positional vertigo

	Frequency		Chi-square
Diseases	Absolute N	Relative %	(p-value)
Mèniére's disease	72	55,4	
Vestibular neuritis	38	29,2	
Vertebrobasilar insufficiency	14	10,8	< 0,001
Other otological conditions	6	4,6	
Total	130	100,0	

Relative to disease progression, 990 patients (77.9%) became asymptomatic or improved with repositioning maneuver therapy. BPPV recurred in 277 treated patients (21.8%) within one year. Four patients (3.2%) did not improve.

## DISCUSSION

The social and demographic data in 1,271 files of BPPV patients revealed that there were more cases from 41 to 60 years and over 60 years. Baloh et al.^3^ found a higher prevalence of BPPV in the sixth decade when the cause was idiopathic, in the fourth and fifth decades when the cause was post-viral, and from the second to the sixth decades in post-trauma patients. In Brandt's^1^ series, BPPV was considered as very common in elderly patients, especially around age 70 years; at this age, 30.0% of cases had presented this disease at least once.

Female subjects predominated in our series (62.8% of cases), similar to other published reports;^2^, ^3^, ^7^ the female to male ratio was 1.6:1.3

Dizziness and positioning nystagmus were found in 81.3% of our BPPV cases. Absence of nystagmus in the Dix-Hallpike maneuver – which occurred in 18.7% of our cases – was also reported by Tirelli et al.^19^, but only in 9.6% of BPPV cases.

The posterior canal was involved more often than the lateral and anterior canals; this finding is similar to other published results.^3^,^6^,^8^ 11, 12 The explanation for this is that the spatial position of the posterior semicircular canal is a more favorable migration site for statoconia from the utricle.^15^

Posterior canal involvement was more frequently unilateral than bilateral in our series. The disease was unilateral in 91.8% of cases of BPPV with dizziness and positioning nystagmus; it was unilateral in 88.2% of cases of BPPV with dizziness and no positioning nystagmus. This finding is comparable with a previous report of unilateral disease in 88.2% of BPPV cases and bilateral disease in 11.8% of such cases.^12^

There was no bilateral involvement of the anterior and lateral canals in our cases. We found no data in the literature to confront this finding.

Right labyrinth involvement was more frequent in our BPPV patients, which was similar to Von Brevern et al.'s^20^ findings; according to these authors, the right labyrinth is involved 1.41 times more often than the left labyrinth, due to the habit of sleeping in right lateral decubitus.

Canalithiasis was found in 97.5% of our cases, which confirms the findings of Korres, Balatsoura^6^ and Ganança et al.:^12^ these authors have stated that canalithiasis is present in most BPPV cases.

BPPV was considered as idiopathic in the majority of cases (74.8%), similar to previously published results.^3^ 7, 8 A post-trauma etiology was seen in 15.0% of our cases, which compared with the rates described by Baloh et al.^3^ (17.9% of cases), and is higher than Katsarkas's^7^ reported 7.0% of cases. Other causes were rare in our series, as previously reported by Baloh et al.^3^

Ménière's disease was associated with BPPV in 5.7% of our cases; the corresponding percentages described by Baloh et al.,^3^ Hughes and Proctor^9^ were 2.1% and 29.8%.

The particle repositioning maneuver was successful in 77.9% of our patients, which became asymptomatic or improved. The effectiveness of this maneuver in the treatment of BPPV patients has previously ranged from 78.0% to 95.0% of cases^6^ 15, 16, 17.

The recurrence rate of BPPV in a one-year follow-up of our patients after successful repositioning maneuvers was 21.8%. Recurrence rates range from 10.0% to 80.0% of treated cases.^18^,^21^ The recurrence rate of BPPV has been estimated at 15.0% per year.^22^

## CONCLUSION

BPPV is more prevalent over the age of 41 years in white female subjects. In most cases it is idiopathic and presents dizziness and positioning nystagmus due to right posterior canal canalithiasis. Ménière's disease is the most common vestibular disease associated with BPPV. Canalith repositioning maneuvers are effective, but recurrences may occur within the first year.
